# Mielopatía por déficit de cobre: serie de casos y revisión de la literatura

**DOI:** 10.7705/biomedica.6687

**Published:** 2023-06-30

**Authors:** Iván Peña, Juan Sarmiento, Cristian Porras, Ximena Cediel, Ana Camargo

**Affiliations:** 1 Fundación Oftalmológica de Santander (FOSCAL), Bucaramanga, Colombia Fundación Oftalmológica de Santander (FOSCAL) Bucaramanga Colombia; 2 Facultad de Ciencias de la Salud, Universidad Autónoma de Bucaramanga, Bucaramanga, Colombia Universidad Autónoma de Bucaramanga Universidad Autónoma de Bucaramanga Bucaramanga Colombia

**Keywords:** enfermedades de la médula espinal, síndromes de mala absorción, ataxinas, anemia, leucopenia, cobre, Spinal cord diseases, malabsorption syndromes, ataxins, anemia, leukopenia, copper

## Abstract

El déficit de cobre puede presentarse como una mielopatía y manifestarse como una ataxia sensorial secundaria a una desmielinización de los cordones posteriores de la médula espinal. Puede acompañarse de citopenias, principalmente anemia y leucopenia.

Se presenta una serie de casos de tres pacientes con mielopatía por déficit de cobre, diagnosticados y manejados desde el año 2020 al 2022 en un hospital universitario de alta complejidad en Colombia.

Dos de los casos eran mujeres. El rango de edad fue entre 57 y 68 años. En los tres casos, los niveles séricos de cobre estaban disminuidos y en dos de ellos, se descartaron diferentes causas de mielopatía que afectan los cordones posteriores de la médula espinal como el déficit de vitamina B_12_, vitamina E y ácido fólico, tabes dorsal, mielopatía por virus de la inmunodeficiencia humana, esclerosis múltiple e infección por el virus linfotrópico humano de tipo I y II, entre otras. Sin embargo, un paciente tenía deficiencia de vitamina B_12_ asociada con de cobre en el momento del diagnóstico de la mielopatía. En los tres casos hubo ataxia sensitiva y en dos, la paraparesia fue el déficit motor inicial.

Se deben incluir siempre la determinación de los niveles de cobre dentro del abordaje diagnóstico de todo paciente con enfermedad gastrointestinal crónica, con diarrea crónica, síndrome de mala absorción o reducción significativa de la ingestión en la dieta, y que desarrolle síntomas neurológicos sugestivos de compromiso de los cordones, ya que se ha reportado que el retraso en el diagnóstico de las mielopatías se asocia con pobres desenlaces neurológicos.

## Abstract (en)

Copper deficiency can present as myelopathy by the manifestation of sensory ataxia, secondary to demyelination of the posterior cords of the spinal cord, accompanied by cytopenia, mainly anemia, and leukopenia.

Case series study of three patients with myelopathy due to copper deficiency, diagnosed and managed from 2020 to 2022 in a highly complex university hospital in Colombia.

Regarding gender, two cases were female patients. The age range was between 57 and 68 years. In all three cases serum copper levels were decreased, and in two of these, different causes of myelopathy affecting the posterior cords of the spinal cord were ruled out, such as vitamin B_12_, vitamin E and folic acid deficiency, tabes dorsalis, myelopathy due to human immunodeficiency virus, multiple sclerosis and infection by the human lymphotropic virus type I and II, among others. However, at the moment of the myelopathy diagnosis, one patient had vitamin B_12_ deficiency associated with copper insufficiency. All three cases presented sensory ataxia, and in two, paraparesis was the initial motor deficit.

The diagnostic approach must include copper levels assessment in every case of patients with chronic gastrointestinal pathology, chronic diarrhea, malabsorption syndrome, or significant reduction in dietary intake; and the development of neurological symptoms that may suggest cord involvement. It has been reported that a delay in diagnosis can lead to poor neurological outcomes.

El cobre es un metal del grupo de los oligoelementos esenciales y está presente en concentraciones variables en los tejidos. Tiene la capacidad de adoptar dos estados en nuestro organismo: el oxidado o cúprico (Cu^2+^) y el reducido o cuproso (Cu^1+^). Este último se encuentra en mayor porcentaje en nuestro organismo, actuando como cofactor en funciones biológicas y como regulador de vías fisiológicas de producción de energía, metabolismo del hierro, captación de radicales libres, respiración mitocondrial, síntesis de elastina, eritropoyesis y leucopoyesis, entre otras ^1^^,^^2^.

Desde las décadas de 1930 y 1940, el cobre ha despertado interés en la investigación médica: los estudios realizados en cadáveres identificaron su concentración tisular, estimando así las necesidades de cobre en la dieta. El cobre es absorbido por el organismo a través de la mucosa intestinal y transportado por la circulación portal ^3^, mediante la ATPasa transportadora de cobre. En el plasma sanguíneo se une a la histidina y a la albúmina, incorporándose a la ceruloplasmina en el hígado, que de ahí es secretada a la circulación sistémica para ser utilizada en diversos procesos fisiológicos ^3^. Es difícil establecer un valor recomendado para la dieta. Sin embargo, en la literatura se han descrito valores que van de 0,6 a 1,44 mg por día ^4^^-^^6^.

La deficiencia de cobre es una causa rara de manifestaciones neurológicas como la mielopatía o la mieloneuropatía, la neuropatía periférica e incluso la neuritis óptica ^7^. Está relacionada con la mala absorción, y las causas quirúrgicas son las más relacionadas con esta deficiencia, como la derivación gástrica con una prevalencia descrita hasta del 9,6 % ^8^, las cirugías del tracto gastrointestinal superior y las gastrectomías.

Además, existen otros factores de riesgo y condiciones asociadas con la deficiencia de cobre, como suplementos de zinc (porque actúa como agente quelante) y síndromes de mala absorción ^9^. Las principales manifestaciones de esta deficiencia son neurológicas, dadas por mielopatía posterior, mieloneuropatía y neuropatía periférica, e incluso neuritis óptica como se mencionó anteriormente ^7^^-^^9^. Sin embargo, también se han descrito con frecuencia manifestaciones hematológicas dadas por anemia macrocítica o normocítica y leucopenia, y con menor frecuencia, trombocitopenia ^10^.

## Presentación de casos

A continuación, se describe una serie de casos de tres pacientes con diagnóstico de mielopatía por déficit de cobre que presentaron inicialmente manifestaciones neurológicas motoras y sensitivas debidas al déficit de este oligoelemento.

### Caso 1

Se trata de una mujer de 58 años que consultó al servicio de neurología de forma ambulatoria en julio de 2020 por debilidad de los miembros inferiores, con diez meses de dolor urente, asociado a nueve meses de parestesias bilaterales y paraparesia espástica progresiva, con incapacidad para la marcha subsecuente.

Como antecedentes presentaba síndrome de mala absorción secundario a enterocolitis actínica como consecuencia de 25 sesiones de braquiterapia, hasta completar 5.000 cGy, por diagnóstico de cáncer de endometrio en el año 2009; asimismo, adenoma tiroideo tóxico en el 2016 con tratamiento definitivo de ablación con yodo e hipotiroidismo posterior a la ablación secundaria y, por último, antecedentes de osteoporosis, déficit de vitamina D y anemia secundaria por déficit de hierro y vitamina B_12_, diagnosticados en el 2016. Esta última no se resolvió a pesar del tratamiento y la corrección de estos déficits. En la consulta, no refirió ningún síntoma ni signo relevante en la revisión por sistemas.

En el examen físico de ingreso presentó signos vitales normales; se evidenció paraparesia, sensibilidad bilateral disminuida en patrón de bota corta, hipopalestesia bilateral, hiperreflexia con clonus aquiliano y reflejo plantar flexor bilateral; signo de *l’hermitte* negativo sin presentar dismetría, ni adiadococinesia, sin lograr la marcha debido a ataxia sensitiva y sin presentar signos de irritación meníngea. El resto del examen físico fue normal.

Al ingreso, en el hemograma se encontró una citopenia doble dada por anemia leve con hemoglobina de 11,1 g/dl y leucopenia (3.050 por mm^3^) con neutropenia leve (1.189 por mm^3^) (cuadro 1).

**Cuadro 1:** Resultados de laboratorio de los pacientes al momento de realizar el diagnóstico de déficit de cobre

Valores de referencia		Paciente 1	Paciente 2	Paciente 3
Hemograma				
	Hemoglobina (12,3-15,3 g/dl)	11,1	8,2	13,2
	Volumen corpuscular medio (80-96 fl)	92,5	94,3	93,2
	Hemoglobina corpuscular media (28-33 pg)	29,8	31,0	30,7
	Leucocitos (4.400-11.300 por mm^3^)	3.050	1.400	5.670
	Plaquetas (150.000-440.000 por mm^3^)	223.000	143.000	275.000
Etiología infecciosa				
	HTLV I/II, anticuerpos totales	Negativo	Negativo	Negativo
	VDRL	No reactivo	No reactivo	No reactivo
	FTA-ABS	Negativo	Positivo	Negativo
Etiología carencial				
	Vitamina B12 (197-771 pg/ml)	358	615	183
	Ácido fólico (4,4-31 ng/ml)	12,4	11,6	20
	Vitamina E (5,2-18,1 mg/L)	6,7	12	10
	Cobre en suero (0,8-1,5 µg/ml)	0,19	0,17	0,2

**Figura 1:**
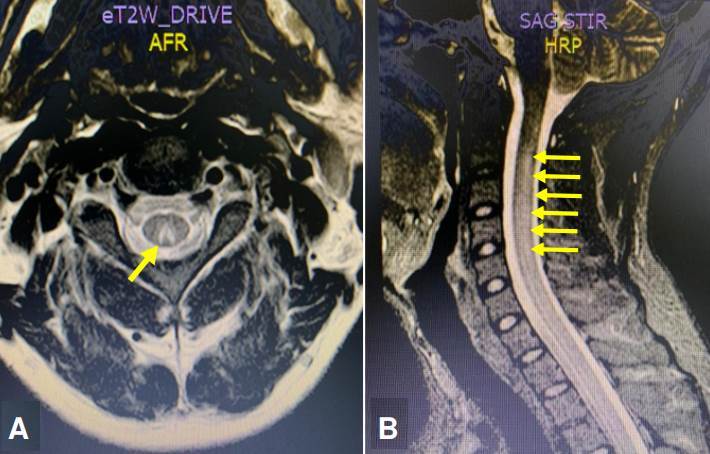
A. Corte axial de RM cervical con gadolinio en secuencia T2 en el que se observa hiperintensidad de los cordones posteriores de la médula espinal cervical, hallazgo descrito como el signo de la V invertida (flecha amarilla). B. Corte sagital de RM de columna cervical y dorsal con gadolinio en secuencia STIR con hiperintensidad de la médula espinal cervical que se extiende hasta la región dorsal (flechas amarillas).

La paciente aportó una electromiografía con conducción nerviosa que reportaba una radiculopatía de la vertebra lumbar L5. Se practicó una resonancia magnética (RM) con gadolinio, de columna cervical y dorsal. Se observó una hiperintensidad que comprometía los cordones posteriores de la médula espinal cervical y dorsal, sin captación del medio de contraste (figura 1). Se sugirió una mielopatía por déficit metabólico de vitamina B_12_, ácido fólico o cobre, o un proceso inflamatorio de tipo infeccioso por virus linfotrópico humano de células T de tipo I o II (HTLV-I/II).

Los potenciales somatosensoriales evocados de miembros superiores fueron normales, los de miembros inferiores no se detectaron. Teniendo en cuenta estos hallazgos, el servicio de neurología consideró que la paciente cursaba con un síndrome de motoneurona superior bilateral relacionado con un síndrome medular posterior y sensitivo bilateral de los cordones. Se analizaron los niveles séricos de vitamina E, vitamina B_12_ y ácido fólico, los cuales fueron normales; además, los anticuerpos totales para HTLV l/ll, la prueba ELISA para virus de la inmunodeficiencia humana y la serología para sífilis (VDRL) resultaron negativos (cuadro 1). Sin embargo, los niveles de cobre estaban disminuidos, por lo que se diagnosticó mielopatía y citopenia doble.

Después de la administración oral de 4 mg al día de cobre elemental y un plan nutricional guiado por un mes, se logró la resolución de la citopenia con valores de hemoglobina de 12,3 g/dl y 5.760 leucocitos por mm^3^. A pesar de ello, se logró una mejoría leve de las manifestaciones neurológicas.

### Caso 2

Se trata de un hombre de 57 años que consultó de forma ambulatoria al servicio de neurología en julio de 2022 por cuadriparesia de tres meses de evolución de forma progresiva, asociada a astenia y adinamia.

Como antecedentes presentaba: infección por HIV desde hace 30 años, en estadio B3, con falla virológica, en manejo antirretroviral con raltegravir asociado a emtricitabina con tenofovir disoproxil fumarato; además, uveítis posterior con desprendimiento de la retina por toxoplasma, tratada farmacológicamente y quirúrgicamente en marzo de 2022; asimismo, pancitopenia (hemoglobina de 8,2 g/dl, 1.400 leucocitos por mm^3^ y 143.000 plaquetas por mm^3^) diagnosticada en abril de 2022, y con etiologías carencial, autoinmunitaria, infiltrativa y neoplásica descartadas; también, refirió sífilis indeterminada en abril de 2022, la cual fue tratada con tres dosis de penicilina benzatínica; y, finalmente, síntomas de desnutrición proteico-calórica desde hace un año por baja ingestión.

En la valoración inicial, los signos vitales se encontraban normales y durante el examen neurológico se documentó cuadriparesia asociada a hiperreflexia, presencia de ataxia sensitiva e incapacidad para la bipedestación y la marcha. El resto del examen físico fue normal.

**Figura 2:**
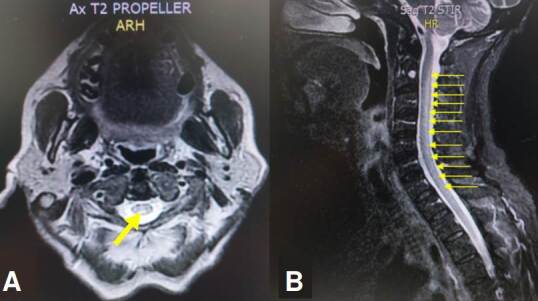
A. Corte axial de RM cervical con gadolinio en secuencia T2 en la que se aprecia hiperintensidad en la región posterior de la médula espinal cervical (flecha amarilla). B. Corte sagital de RM de columna cervical y dorsal con gadolinio en secuencia STIR en el que se evidencia hiperintensidad que compromete la médula espinal cervical y dorsal en su totalidad (flechas amarillas).

Se ordenó RM cervical y dorsal con imagen de hiperintensidad lineal en el aspecto posterior central de los cordones dorsales que comprometía toda la médula espinal cervical (C1 a C6) y dorsal (T1 a T12) (figura 2 ).

Por el antecedente de sífilis indeterminada tratada se realizó punción lumbar, análisis citoquímico y citológico de líquido cefalorraquídeo, y estudios infecciosos (VDRL, prueba de KOH, tinción negativa con nigrosina, tinción de Gram, antígeno para criptococo, baciloscopia, cultivo para gérmenes comunes y para micobacterias), todos con resultado negativo. Se descartó neurosífilis y mielopatía por HTLV I/II.

Los niveles de vitamina B_12_, vitamina E y ácido fólico fueron normales. Sin embargo, los niveles de cobre sérico se encontraron disminuidos (cuadro 1), por lo cual se diagnosticó mielopatía por déficit de cobre y se inició suplemento oral de 4 mg de cobre elemental al día.

En el seguimiento de los tres meses, se evidenció una leve mejoría de los síntomas neurológicos, con ligero aumento de la fuerza en los miembros superiores y una resolución completa de la pancitopenia con valores de hemoglobina de 14 g/dl, 4.270 leucocitos por mm^3^ y 229.000 plaquetas por mm^3^.

### Caso 3

Se trata de una mujer de 68 años que consultó al servicio de neurología en marzo de 2022 por paraparesia de seis meses de evolución, de carácter progresivo, asociada a disminución de la sensibilidad en patrón de bota corta.

Como antecedente relevante refirió diarrea crónica sin sangre ni moco, de un año de duración y secundaria a la realización de una colecistectomía, debido a colelitiasis, previamente tratada con colestiramina. La revisión por sistemas no sugirió ninguna alteración relevante.

En el examen físico, los signos vitales normales fueron normales; se encontró paraparesia espástica y sensibilidad bilateral disminuida en patrón de bota corta, hipopalestesia bilateral sin dismetría ni adiadococinesia, con incapacidad para la marcha por ataxia y prueba de Romberg positiva.

**Figura 3:**
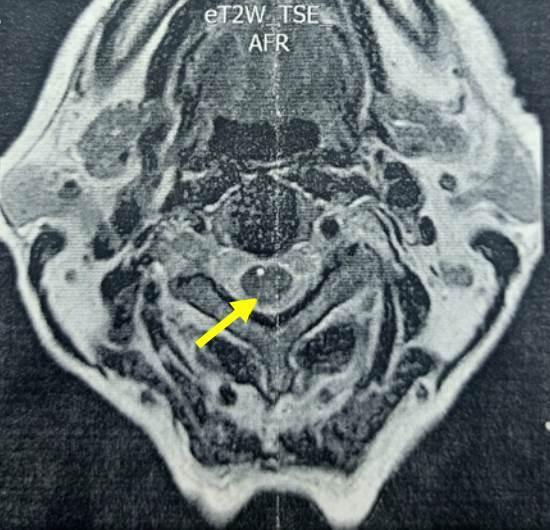
Corte axial de RM cervical con gadolinio en secuencia T2 en la que se observa hiperintensidad de los cordones posteriores de la médula espinal cervical (flecha amarilla).

En los resultados de la RM con contraste de columna cervical y dorsal se apreciaron hiperintensidades en la región posterior de la médula espinal desde la C1 hasta T1 (figura 3), en el contexto de una mielopatía de los cordones posteriores.

Las pruebas serológicas para VIH, sífilis, infección por HTLV I/II fueron negativas. Sin embargo, los niveles de vitamina B_12_ y de cobre sérico estaban acentuadamente disminuidos (cuadro 1).

Se diagnosticó mielopatía de los cordones secundaria a estos déficits y se inició tratamiento. Después de recibir reposición parenteral de vitamina B_12_ y suplemento oral de 4 mg de cobre al día por dos meses presentó una mejoría neurológica leve.

## Discusión

La deficiencia de cobre se ha definido como un nivel sérico de cobre inferior a 0,8 µg/dl, asociado a un valor de ceruloplasmina menor de 20 mg/dl. En la mayoría de los casos, esto se debe a condiciones que generan reservas metabólicas insuficientes de cobre como síndromes de mala absorción por cirugías bariátricas ^1^. En un estudio retrospectivo de 40 pacientes con anormalidades hematológicas y déficit de cobre se reportó una prevalencia del 25 % en pacientes sometidos a cirugía bariátrica y del 35 % en pacientes sometidos a cirugía gastrointestinal en general ^11^.

La revisión sistemática de Kumar *et al*., publicada en 2016, describió el déficit de cobre hasta en el 10 % en pacientes sometidos a cirugía de derivación gástrica o en Y de Roux ^12^. Los estudios de Pellitero y colaboradores, y de Gobato y colaboradores, reportaron un aumento en la prevalencia del déficit del oligoelemento a los seis y doce meses de seguimiento en pacientes sometidos a derivación gástrica y gastrectomía en manga, respectivamente. No se encontraron estudios describiendo el valor del cobre sérico ^13^.

Asimismo, el déficit de cobre ha sido descrito en pacientes con enteropatías ^1^, como en los casos número 1 y 3. No obstante, también se han descrito como causas del déficit, la ingestión insuficiente de cobre y la excesiva de zinc, debido a que este último actúa como quelante del cobre impidiendo su absorción a nivel intestinal ^1^. Por otra parte, en poblaciones vulnerables, afectadas por la desnutrición y la pobreza, la ingestión insuficiente del oligoelemento ha sido descrita como la principal causa de esta deficiencia ^1^, lo cual podría explicar el déficit en el paciente número 2, quien presentaba el antecedente de desnutrición proteico-calórica por una dieta baja en calorías y oligoelementos; además, no tenía antecedentes de cirugías bariátricas, ni de enteropatías o síndrome de malabsorción, ni de ingestión de zinc.

Este déficit también puede deberse a una mayor demanda del oligoelemento, como ocurre durante el embarazo o la lactancia, entre otros, o a la presencia de enfermedades hereditarias como la enfermedad de Menkes ^1^^,^^14^. Otras causas descritas son la nutrición entérica o parenteral en pacientes con enfermedades que impiden la alimentación natural. Por ejemplo, en los casos de nutrición por yeyunostomía, anatómicamente se genera un salto en el sitio de absorción, lo que provoca una presentación similar a la de los síndromes de mala absorción, como en la enfermedad celiaca, la enfermedad de Crohn o el síndrome del intestino corto ^1^.

Sin embargo, como se mencionó anteriormente, la mayoría de los casos de deficiencia de cobre se han relacionado con mala absorción secundaria a procedimientos quirúrgicos gastrointestinales como la cirugía bariátrica, los procedimientos de cruce duodenal y los de derivación gástrica ^15^^-^^17^.

En cuanto a su presentación, en una serie de siete casos publicada en el 2018, se encontró que la edad media fue de 57,4 años ^14^, lo cual concuerda con los casos 1 y 2. En la misma serie, los pacientes manifestaron como primer síntoma, un trastorno de la marcha dado por ataxia sensitiva progresiva, pérdida de la propiocepción y sensación vibratoria de ambos pies; también, una alteración en la discriminación del dolor y la temperatura en las extremidades inferiores relacionada con un síndrome de los cordones posteriores ^14^. Estos hallazgos concuerdan con lo descrito en nuestros casos.

Poujois y colaboradores reportaron que el 70 % de los pacientes presentaba parestesias ascendentes y el 50 % de ellos, signos y síntomas de síndrome de los cordones laterales ^14^. No obstante, cabe resaltar que, a pesar de que la principal manifestación descrita relacionada con el déficit de cobre es la mielopatía, como ocurrió en los tres pacientes de nuestra serie, también puede manifestarse por mieloneuropatía, neuropatía periférica y excepcionalmente como una neuropatía óptica ^8^^,^^9^. Otros hallazgos de Poujois *et al*. describen anemia normocítica o macrocítica en el 85,7 % de los pacientes, así como reportamos en los casos número 1 y 2, y también reportaron linfopenia en el 71,4 % de los casos ^14^.

Otra serie de seis pacientes del 2009, que buscaba describir las alteraciones clínicas y electrodiagnósticas en pacientes con mieloneuropatía por déficit de cobre, encontró anemia asociada a leucopenia en todos los casos. En el contexto de un síndrome mielodisplásico, esta citopenia doble se presentó en el 50 % de los pacientes y se resolvió tras la suplementación con cobre ^18^. Otros estudios observacionales, como el caso publicado por Wazir y Ghobrial, han descrito la triada de anemia, leucopenia y mieloneuropatía en un mismo paciente ^19^.

Respecto a las etiologías metabólicas que comprometen la región posterior de la médula espinal, especialmente en la columna dorsal, se han descrito principalmente el déficit de vitamina B_12_, vitamina E, cobre y ácido fólico. La mielopatía de los cordones posteriores tiene un cuadro clínico muy bien establecido. En los pacientes con HIV, cáncer y cirugía gastrointestinal es probable encontrar más de una etiología, lo que se debe tener en cuenta al hacer el enfoque diagnóstico. El déficit de vitamina B_12_ o cobalamina es la causa más conocida de mielopatía metabólica y también puede comprometer, en un grado menor, la región lateral de la médula espinal. En la RM se vería en la secuencia T2 una hiperintensidad bilateral en la columna dorsal de la región cervical y torácica de la médula espinal, formando el signo de la V invertida ^20^.

Típicamente no existe captación del medio de contraste, sin embargo, posterior a su administración, se podría ver el realce de la región posterior y lateral de la médula espinal en la secuencia T1 ^21^. Estos hallazgos imagenológicos y clínicos son indistinguibles a los que se presentan en la mielopatía por déficit de cobre ^20^. De igual forma, hay que resaltar que en la secuencia STIR (*Short Time Inversión Recovery*) se observa usualmente una hiperintensidad en la mayoría de las mielopatías ^21^, como en las imágenes de RM presentadas en los casos 1 y 2.

Por otra parte, la exposición a óxido nítrico ya sea como anestésico inhalado durante procedimientos dentales o como medicamento de abuso, puede causar deficiencia funcional de vitamina B_12_. Esto afecta la región dorsal de la médula espinal, ya que esta molécula oxida el átomo de cobalto necesario para el correcto funcionamiento de la cobalamina ^20^. Igualmente, la ingestión excesiva de zinc puede comprometer la región dorsal de la médula espinal, debido a que produce deficiencia de cobre, pues el zinc estimula la unión de iones de cobre en el tracto gastrointestinal, lo cual previene su absorción adecuada ^20^.

El déficit de ácido fólico también puede causar mielopatía, neuropatía periférica, atrofia óptica y problemas cognitivos. Sin embargo, este déficit ocurre con menor frecuencia que el de cobalamina y usualmente está asociado con otras deficiencias nutricionales ^22^^,^^23^.

En cuanto a la deficiencia de vitamina E, esta se asocia con mayor frecuencia a síndromes de mala absorción, pero también puede ser una consecuencia de mutaciones en la proteína de transferencia del alfa tocoferol, en la proteína de transferencia de triglicérido microsómico causando abetalipoproteinemia, o defectos en la síntesis y secreción de quilomicrones. Aunque clínicamente puede generar alteración en la vibración y propiocepción por compromiso de los cordones posteriores de la médula espinal, el déficit de vitamina E puede ocasionar síndromes espinocerebelosos y neuropatías, teniendo como hallazgos principales en la RM, la hiperintensidad de los cordones posteriores en la secuencia T2 con atrofia cerebelar o sin ella ^22^.

El compromiso de la región dorsal de la medula espinal puede darse también por etiologías paraneoplásicas de los tumores de pulmón (especialmente el carcinoma de células pequeñas), mama, timo y ovario; o por etiologías autoinmunológicas e infecciosas como la tabes dorsal ^20^.

En cuanto al déficit de vitamina B_12_, en los casos número 1 y 2 se descartó, pero en el tercer caso sí se documentó deficiencia de cobalamina asociada a deficiencia de cobre: un déficit mixto ya descrito ^24^, que eventualmente podría contribuir al desarrollo de una mielopatía. De igual forma, como se describió previamente, otras condiciones como la exposición a óxido nítrico, la ingestión excesiva de zinc, las etiologías neoplásicas, autoinmunes e infecciosas -incluyendo la mielopatía por HTLV-I/II y la tabes dorsal- fueron descartadas en los tres casos con base en la historia clínica y en los exámenes diagnósticos practicados (cuadro 1).

No existen lineamientos específicos que indiquen cómo debe ser tratada esta condición. Sin embargo, las diversas revisiones narrativas y los reportes de casos sugieren la reposición de cobre por vía oral o endovenosa, refiriendo que, en pacientes con síntomas neurológicos, una dosis de 2 a 4 mg de cobre elemental diario es suficiente ^17^. Este fue el esquema utilizado en los tres casos descritos. También se ha recomendado el esquema de 8 mg diarios durante una semana, seguido de 6 mg diarios durante una semana, luego 4 mg diarios durante una semana y, finalmente, 2 mg diarios ^23^. La guía de práctica clínica de la Sociedad Americana de Medicina Metabólica y Bariátrica recomienda suministrar de 2 a 4 mg de cobre intravenoso por seis días, seguido de 3 a 8 mg de cobre oral hasta que los niveles se normalicen ^25^.

Respecto al pronóstico, las manifestaciones hematológicas como la anemia y la leucopenia se resuelven en tres meses, aproximadamente ^1^, como sucedió en los casos número 1 y 2. No obstante, la recuperación de los síntomas neurológicos es variable, y se presentan déficits neurológicos residuales en la mayoría de los casos.

En la actualidad, se reporta únicamente mejoría en los estudios de conducción nerviosa, potenciales evocados y hallazgos en la RM. En los tres casos descritos hubo una leve mejoría de la paresia, tras varios meses con suplemento oral de cobre. Sin embargo, los pacientes persistieron con ataxia significativa que les impide la marcha independiente. Finalmente, se ha descrito que el pronóstico de los síntomas neurológicos está influenciado por la gravedad y la duración previa del déficit de cobre ^1^.

## Conclusión

Se ha descrito que el retraso en el diagnóstico de mielopatías está asociado con pobres desenlaces neurológicos. Por lo anterior, debe incluirse el análisis de los niveles de cobre en el abordaje diagnóstico de todo paciente con patología crónica gastrointestinal, diarrea crónica, síndrome de mala absorción o reducción significativa de la ingestión y que tenga síntomas neurológicos sugerentes de compromiso de los cordones nerviosos.
